# Diabetes mellitus and risk of multiple sclerosis: a systematic review and meta-analysis

**DOI:** 10.3389/fendo.2026.1724167

**Published:** 2026-02-18

**Authors:** Maoqin Tang, Hongxue Tang, Haoyue Deng, Ruihan Liu, Gongjing He

**Affiliations:** 1 Department of Rehabilitative Medicine, Shapingba Hospital, Chongqing University (Shapingba District People’s Hospital, Chongqing), Chongqing, China; 2 Department of Oncology, Army Medical Center of PLA, Chongqing, China; 3 Department of Scientific Research, Shapingba Hospital, Chongqing University (Shapingba District People’s Hospital, Chongqing), Chongqing, China

**Keywords:** cohort studies, diabetes mellitus, meta-analysis, multiple sclerosis, risk of disease

## Abstract

**Background:**

Multiple studies have suggested a potential association between multiple sclerosis (MS) and diabetes mellitus (DM). However, inconsistencies in the published literature—attributable to variations in statistical methods, sample size, and study design—have led to conflicting conclusions. Therefore, we conducted a systematic meta-analysis to clarify the relationship between MS and DM and to inform future mechanistic research.

**Methods:**

We performed a comprehensive literature search in the Web of Science, PubMed, and Embase databases for studies investigating the relationship between MS and DM, covering the period from database inception to July 2025. Two researchers independently screened studies, extracted data, and assessed the risk of bias. Meta-analyses were conducted using Review Manager 5.3.

**Results:**

Seven studies involving 3,143,619 participants were included. The meta-analysis did not reveal a statistically significant overall association between MS and DM (HR = 1.05,95%CI:0.88-1.27, *P* = 0.58). However, DM was associated with an increased risk of developing MS (HR = 1.59, 95%CI:1.24-2.05, *P* = 0.0003). Furthermore, patients with type 2 diabetes mellitus (T2DM) also showed an elevated risk of MS (HR = 1.46,95%CI:1.10-1.94, *P* = 0.008).

**Conclusions:**

Current evidence indicates that DM, specifically T2DM, increases the risk of developing MS. However, a reverse association remains unconfirmed.

## Introduction

Multiple sclerosis (MS) is an immune-mediated inflammatory demyelinating disease of the central nervous system. Globally, it is estimated that 2.8 million people are living with MS. The female-to-male ratio is approximately 3:1, with a median age of onset at 32 years (1). The prevalence of MS varies significantly across countries and regions, a difference largely attributed to geographic latitude and its association with vitamin D exposure (2). The highest prevalence rates are observed in Western Europe and North America, followed by Central and Eastern Europe, the Balkan Peninsula, Australia and New Zealand. The lowest rates are found in Asia, Africa and the Middle East (3, 4). The pathogenesis of MS involves autoimmune dysregulation, where activated immune cells erroneously attack the myelin sheath, disrupting nerve conduction (5). This poses a serious threat to neurological function and quality of life, particularly among young and middle-aged adults.

Diabetes mellitus (DM) represents one of the most pressing global health challenges. In 2023, the overall prevalence of DM in China reached 13.7%, affecting approximately 233 million individuals - a 163% increase since 2005 (6, 7). Without effective intervention, nearly 30% of Chinese adults could be affected by DM by 2050 (6). Worldwide in 2024, 589 million adults aged 20–79 were living with DM, a number projected to rise to 853 million by 2050 (8). The intensifying trends of population aging and lifestyle changes are contributing to a growing disease burden attributable to DM. Based on etiology and pathogenesis, DM is primarily classified into several types, the most common being type 1 (T1DM) and type 2 (T2DM) (7). T1DM is a chronic autoimmune disease characterized by the destruction of insulin-producing pancreatic β-cells, leading to absolute insulin deficiency (9). As the most prevalent subtype globally, T2DM is defined by core features of insulin resistance coupled with dysfunctional pancreatic β-cells (10).

Both DM and MS are chronic conditions involving immune system dysregulation (11). In clinical practice, the co-occurrence of MS and DM is increasingly observed. Epidemiological studies indicate that the risk of developing DM is significantly higher in patients with MS than in the general population, and this comorbidity rate continues to rise (12). Conversely, the risk of MS is also elevated among individuals with DM (13, 14). Population-based cohort studies have demonstrated a significant association between DM and an increased risk of MS (15), and the prevalence of DM is higher in MS patients compared to non-MS individuals (15, 16). One such study further confirmed that DM is associated with an elevated risk of developing MS (17). When both conditions coexist, patients face not only the neurological deficits caused by MS but also the metabolic disturbances triggered by DM, resulting in a more complex and challenging clinical presentation.

In the context of T2DM, systemic effects extend to the central nervous system, impairing brain insulin signaling, inhibiting the differentiation of oligodendrocyte precursor cells, and reducing myelination (18). Persistent hyperglycemia impacts myelin metabolism through multiple pathways: first, by inducing oxidative stress that promotes lipid peroxidation in myelin, compromising its structural integrity (19); second, by accelerating the formation of advanced glycation end products (AGEs), which alter myelin protein structure and function upon binding while activating pro-inflammatory pathways (20); and third, by causing energy deficiency in oligodendrocytes, thereby hindering myelin synthesis (21). Furthermore, dyslipidemia associated with T2DM interferes with central nervous system function (22): disrupted cholesterol metabolism impairs the ability of oligodendrocytes to build myelin (23), and sphingolipid imbalance leads to the accumulation of neurotoxic species, damaging oligodendrocytes and inducing apoptosis (24). T2DM may also elevate the risk of MS through genetic susceptibility (25, 26) and environmental factors such as viral infections (27). In contrast, the link between T1DM and MS has a clearer immunogenetic basis (28). Both diseases share genetic risks primarily located within the human leukocyte antigen (HLA) class II region (29). Certain single-nucleotide polymorphisms in immune-related pathways are also associated with this comorbidity, and overlapping immune features exist between the two conditions, although the probability of sharing fully pathogenic haplotypes is relatively low (30). This suggests that both T1DM and MS are polygenic disorders resulting from the interaction between genetic predisposition and environmental factors (29).

The evidence summarized above indicates a potential correlation between DM and MS. However, the nature of their connection and the bidirectional relationship between these diseases remain insufficiently clarified. Therefore, we conducted a meta-analysis of bidirectional associations to systematically explore the interrelationship between MS and DM.

## Methods

This meta-analysis was conducted in accordance with the latest version of the Preferred Reporting Items for Systematic Reviews and Meta-Analyses (PRISMA) guidelines (31–33).

### Search strategy

A systematic literature search was performed in the Web of Science, PubMed, and Embase electronic databases to identify studies investigating the interrelationship between MS and DM. The search covered the period from database inception to July 2025. We employed a combination of Medical Subject Headings (MeSH) terms and free-text keywords. The reference lists of eligible articles were also manually screened to identify additional relevant studies. Key search terms included “multiple sclerosis,” “diabetes mellitus,” “association,” and “risk.” The detailed search strategy for PubMed is provided in **Supplementary Material Table S1**.

### Eligibility criteria

#### Inclusion and exclusion criteria

Studies were selected based on the following PICOS framework:

P (Population): Patients with a confirmed clinical diagnosis of MS or DM;

I (Intervention): Diagnosis of DM (for assessing MS risk) or diagnosis of MS (for assessing DM risk).

C (Comparison): Individuals without DM (for MS risk studies) or individuals without MS (for DM risk studies).

O (Outcomes): Reported effect estimates (e.g., hazard ratio, risk ratio) with corresponding 95% confidence intervals (CIs) for the association between MS and DM.

S (Study Design): Cohort studies (prospective or retrospective).

#### Exclusion criteria

1. Duplicate publications or studies using overlapping data sources.
2. Secondary research literature (e.g., reviews, meta-analyses, systematic reviews).
3. Studies lacking a control group or with a control group that did not meet the predefined criteria.
4. Studies with insufficient data or where the full text was unavailable.

### Data extraction

Two investigators independently screened the titles, abstracts, and full texts of identified records against the eligibility criteria. Any discrepancies were resolved through discussion or by consultation with a third reviewer. Data from included studies were extracted into a standardized Excel spreadsheet. Extracted information included: first author, publication year, country, study design, sample size, participant age, follow-up duration, diagnostic criteria for MS/DM, adjusted variables, effect size estimates with 95% CIs, and risk of bias assessment results.

### Biased risk assessment

The methodological quality of included cohort studies was assessed independently by two reviewers using the Newcastle-Ottawa Scale (NOS). The NOS evaluates three domains: selection of study groups, comparability of groups, and ascertainment of exposure/outcome. Studies scoring 7–9 points were classified as high quality, 4–6 as medium quality, and below 4 as low quality. Disagreements in scoring were resolved by consensus or with input from a third reviewer.

### Data synthesis and analysis

All statistical analyses were performed using Review Manager (RevMan) software version 5.3. The association between DM and MS was quantified using hazard ratios (HRs) with 95% CIs. Heterogeneity across studies was assessed using Cochran’s Q test and the I² statistic, with a significance level set at α = 0.10. An I² value ≤ 50% and a *P*-value > 0.10 indicated low heterogeneity, in which case a fixed-effects model was applied; otherwise, a random-effects model was used. Sensitivity analysis was conducted by sequentially excluding each study to evaluate the robustness of the pooled results. Publication bias was assessed visually using funnel plots.

## Results

### Study characteristics

**Figure 1:**
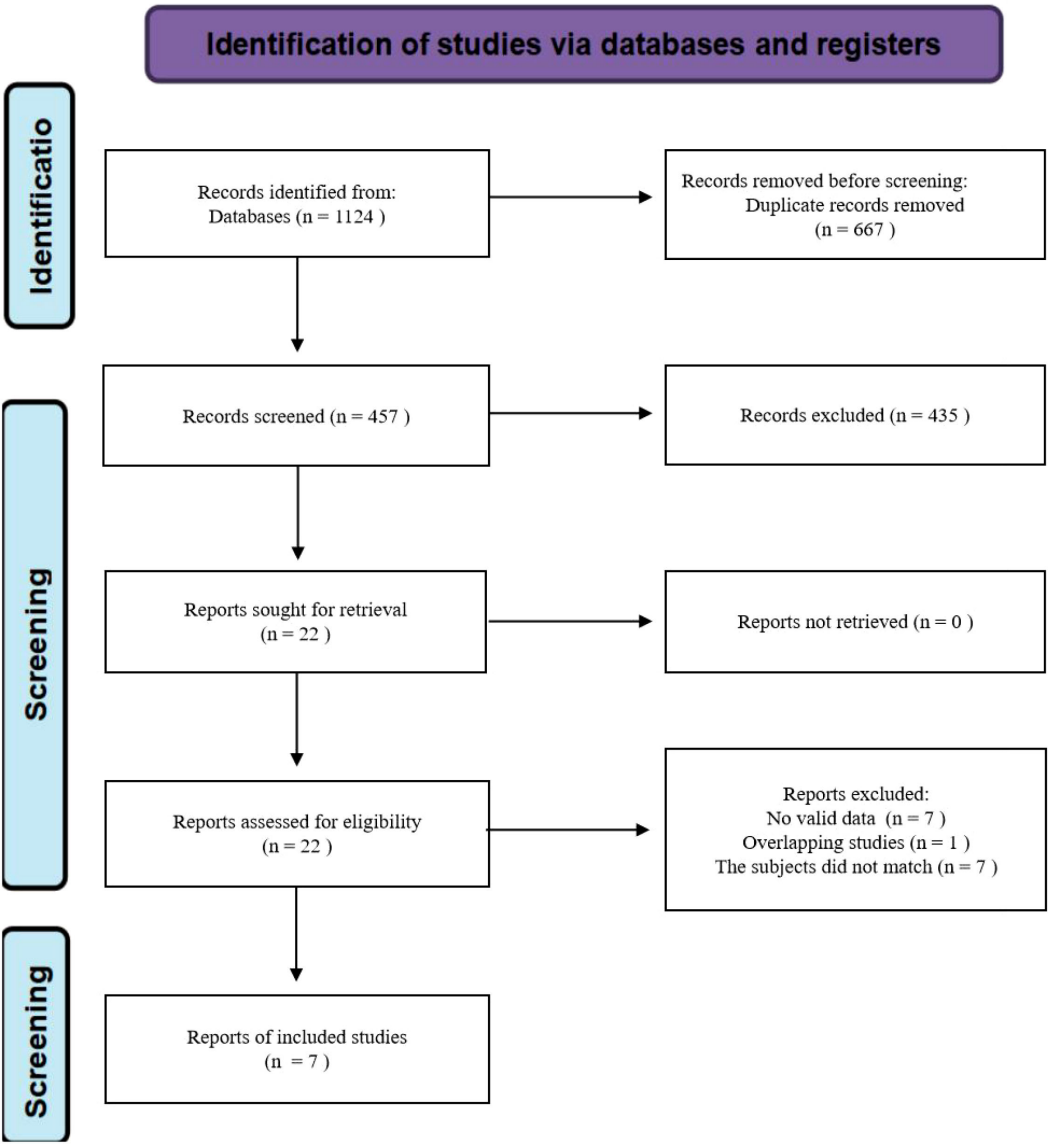
Flowchart of database search and study inclusion.

A total of 1,124 relevant records were identified through database searches. After removing 667 duplicates, 457 records remained for title and abstract screening, which led to the exclusion of 435 articles. Following full-text review of the remaining 22 articles, 15 were excluded based on the predefined criteria. Finally, seven studies were included in the meta-analysis (**Figure 1**). All included studies were cohort designs, published between 2010 and 2024. The total sample size across studies was 3,143,619 participants. The methodological quality assessed by the NOS ranged from 6 to 8 points (**Supplementary Material Table S2**). The basic characteristics of the included studies are summarized in **Table 1**.

**Table 1:** Study characteristics.

Author, year	Country	Study design	No. of patients	Age (median/mean)	Follow-up	Diagnostic criteria	Adjustment
Kang et al., 2010 (49)	China	Cohort study	5388	NR	NR	ICD-9	Gender, age, and socioeconomic status
Hou et al., 2017 (15)	China	Cohort study	1228644	NR	NR	ICD-9	Age, `sex, income-based insurance premium, geographic area of living, urbanization status of living area, Charlson Comorbidity Index, frequency of medical visit, and selected co-morbidities, including allergy, Alzheimer’s disease, anterior horn cell disease, COPD, obesity, vitamin D deficiency, dyslipidemia, hypertension, inflammatory bowel disease, spinal cord injury, stroke, thyroid disease, and traumatic brain injury, anxiety, depression, stress, tonsillectomy, and appendectomy.
Jacobsen et al., 2024 (50)	Denmark	Cohort study	80256	42	19, years	ICD-10	Age, sex, year of diagnosis, marital status, and comorbidities such as IHD, AF, COPD, rheumatoid arthritis, thyroid disease, mononucleosis, IBD, cancer, hypertension, and stroke.
Palladino et al., 2024 (46)	UK	Cohort study	84823	44.9	30, years	ICD-X	Gender, ethnicity, region, deprivation index, number of primary care visits, smoking status, and electronic frailty index.
Cho et al., 2024 (45)	South Korea	Cohort study	10806	NR	MS vs controls: 4.5 vs 4.8 years	ICD-10	Age, sex, income, hypertension, dyslipidemia, stroke, myocardial infarction, congestive heart failure, end-stage renal disease, cancer, and steroid use within 1 year.
Nielsen et al., 2021 (13)	Denmark	Cohort study	1633436	NR	NR	ICD-8, ICD-10	Adjusted for age, sex, birth cohort (1year intervals 1978–1998 and ≧̸1999), as well as for maternal age (<20 20–29, 30–39, ≧̸40years), paternal age (<20, 20–29, 30–39, ≧̸40years), and maternal obesity.
Brnabic et al., 2024 (51)	America	Cohort study	100266	NR	6, years	NR	Age, sex, region of residence, index year and payer type. Baseline clinical characteristics assessed during the pre-index period were hypertension, hyperlipidaemia, tobacco use, use of mobility aidsand chronic disease burden using the Charlson Comorbidity Index. Duration of patients’ follow-up period, discontinuation of index drug and time to drug discontinuation were also examined.

UK, United Kingdom; NR, Not report; MS, Multiple Sclerosis; ICD, International Classification of Diseases; IHD, Ischemic heart disease; COPD, chronic obstructive pulmonary diseases; IBD, Inflammatory bowel disease.

### Risk of T2DM in patients with MS

**Figure 2:**
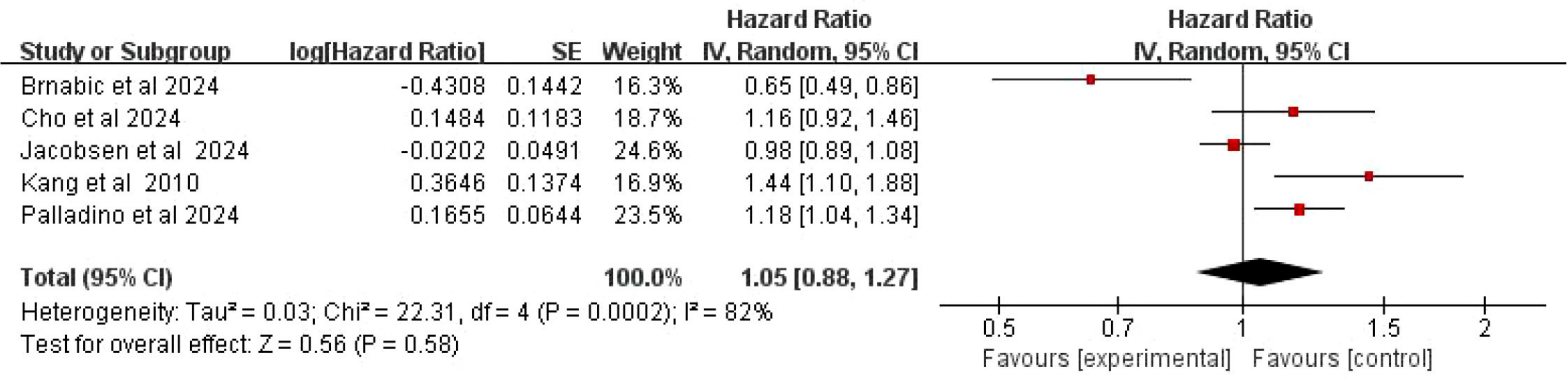
Forest plot of the risk of T2DM in patients with MS.

Five studies reported data on the risk of T2DM in patients with MS. Significant heterogeneity was observed among these studies (*I^2^* = 82%, *P* = 0.0002); therefore, a random-effects model was applied. The meta-analysis indicated no statistically significant difference in the risk of developing T2DM among MS patients (HR = 1.05, 95%CI:0.88-1.27, *P* = 0.58) (**Figure 2**).

### Risk of MS in patients with DM

**Figure 3:**
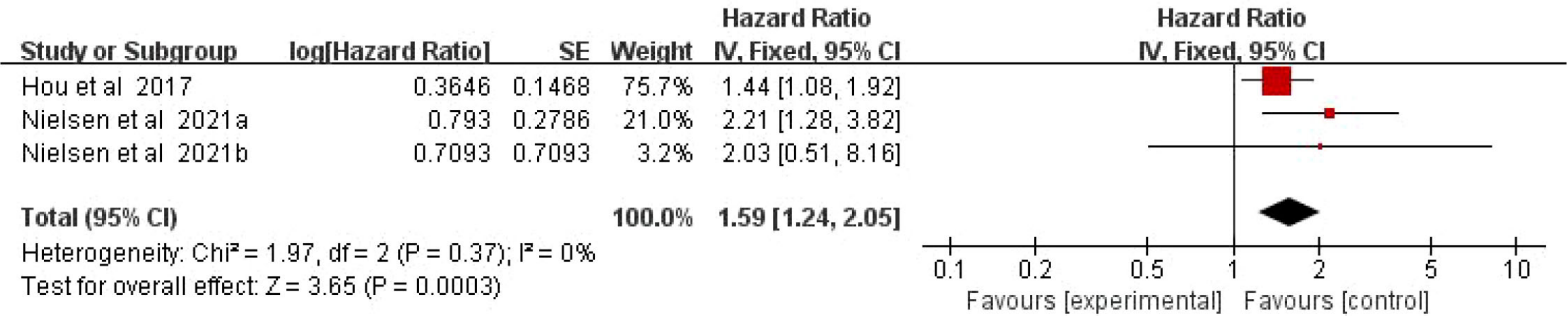
Forest plot of the risk of MS in patients with DM.

Two literature reports on the risk of MS in patients with DM. No significant heterogeneity was detected (*I^2^* = 0%, *P* = 0.37), allowing for the use of a fixed-effect model. The meta-analysis demonstrated a significantly increased risk of developing MS among patients with DM (HR = 1.59, 95%CI:1.24-2.05, *P* = 0.0003) (**Figure 3**).

### Sensitivity analysis

**Figure 4:**
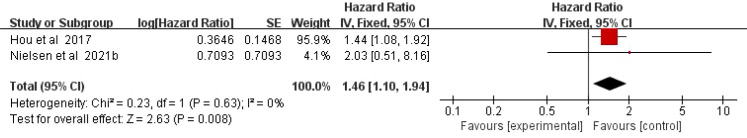
Forest plot of the risk of MS in patients with T2DM.

A sensitivity analysis was performed by sequentially excluding each study. The overall pooled results remained stable regardless of which study was omitted. A secondary analysis focusing specifically on T2DM patients - excluding data pertaining to T1DM-also showed a significant association, with an increased risk of MS (HR = 1.46, 95%CI:1.10-1.94, *P* = 0.008) (**Figure 4**).

### Publication bias

**Figure 5:**
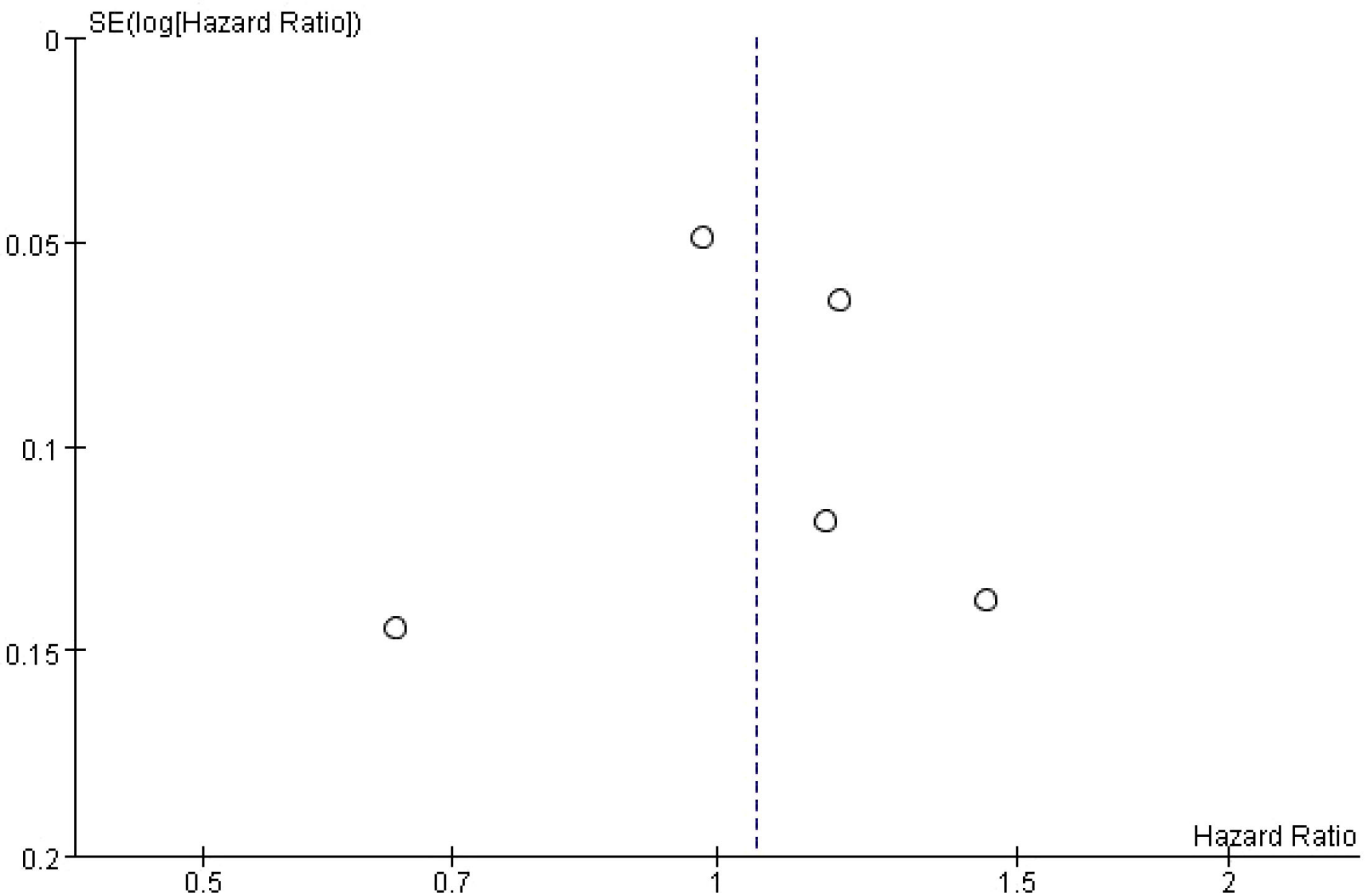
Funnel plot of the risk of T2DM in patients with MS.

**Figure 6:**
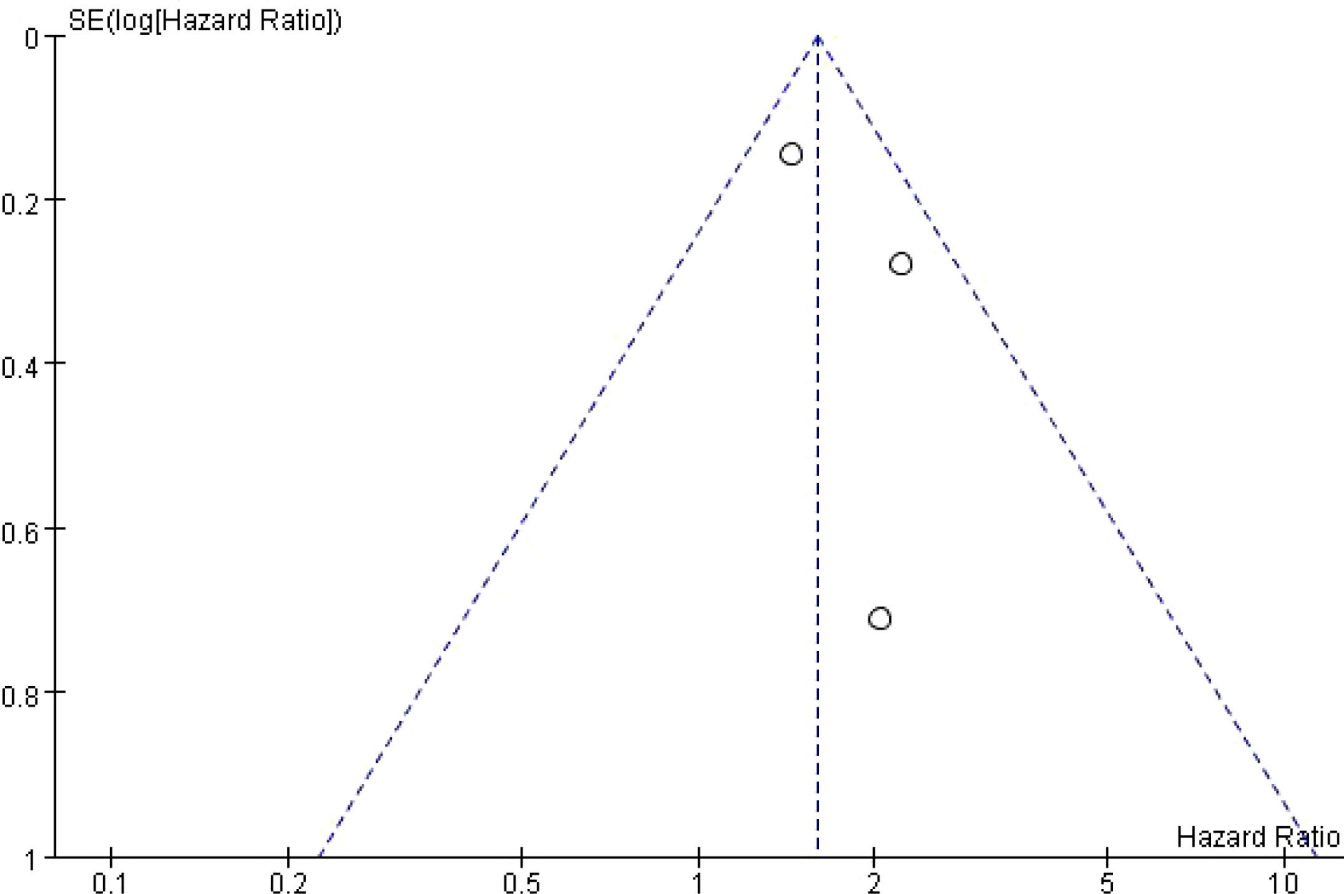
Funnel plot of the risk of MS in patients with DM.

Funnel plots were generated to assess potential publication bias. Visual inspection revealed that the points were distributed in a roughly symmetrical pattern on both sides of the funnel, suggesting no significant publication bias (**Figures 5**, **6**).

## Discussion

This systematic review and meta-analysis investigated the potential association between DM and multiple sclerosis MS. The primary finding indicates that DM significantly increases the risk of developing MS. This provides evidence-based support for a deeper understanding of the interplay between these two conditions. Although the overall bidirectional risk association between MS and DM was not statistically significant, the observed link from DM to MS suggests that metabolic dysregulation may play a significant role in MS pathogenesis. Chronic inflammation, immune system imbalance, and vascular complications associated with DM may collectively affect central nervous system homeostasis, thereby potentially elevating MS risk.

From a mechanistic perspective, MS is a central nervous system autoimmune disease (34). Its core pathology involves an imbalance in CD4+ T-cell subsets, leading to immune-inflammatory infiltration that attacks oligodendrocytes and results in demyelination (35). This process is driven by an interplay of genetic factors, such as HLA class II gene variants, and environmental triggers like viral infections (36, 37). The pathogenesis of DM subtypes, however, differs significantly. T1DM is also an autoimmune disorder, characterized by autoimmune destruction of pancreatic β-cells by autoreactive T cells, leading to absolute insulin deficiency. T1DM shares an immunogenetic background with MS—including HLA haplotypes and immune gene polymorphisms—which forms an important basis for their co-occurrence (28, 30). In contrast, T2DM is centered on insulin resistance and β-cell dysfunction. While lacking direct autoimmune targets, T2DM induces chronic low-grade inflammation and immune dysregulation. Through hyperglycemia and dyslipidemia, it can promote oxidative stress, accumulation of AGEs, and metabolic inflammation, potentially damaging oligodendrocytes, disrupting myelin metabolism, and exacerbating central nervous system inflammation, thereby intersecting with MS pathology T2DM (38). This offers a plausible pathway explaining how T2DM may increase MS risk. In summary, the association between T1DM and MS is founded on a shared immunogenetic basis, whereas T2DM likely contributes to MS pathogenesis primarily through metabolic-inflammatory pathways.

Research indicates that among children and adolescents with DM, the risk of developing MS is significantly elevated (39). A study by Hou et al. further reports an increased MS risk in both male and female patients with T2DM (15), with the risk being particularly high in female patients aged 50 years and younger. This aligns with our finding that DM significantly elevates MS risk. Patients with DM often exhibit a state of chronic low-grade inflammation, marked by significantly elevated serum levels of inflammatory factors such as interleukin-6 (IL-6), tumor necrosis factor-α (TNF-α), and C-reactive protein (40). This systemic inflammation may disrupt immune balance within the central nervous system and potentially trigger MS onset. Additionally, DM can cause immune system dysfunction, impairing the normal activity of T cells and B cells, which may increase susceptibility to autoimmune diseases like MS. Vascular complications of DM may lead to cerebral microcirculatory disturbances, affecting normal myelin metabolism and repair, thus contributing to MS risk. Furthermore, hyperglycemia and insulin resistance can impair neuronal energy metabolism. Insulin resistance weakens nutrition and energy-related signaling mediated by insulin, severely disrupting neurometabolism and leading to neuronal injury (41). Hyperglycemia-induced activation of protein kinase C affects vascular endothelial growth factor expression, promoting vasoconstriction and hypoxia (42, 43). DM is also associated with reduced levels of neurotrophic and angiogenic factors, contributing to neuronal dysfunction (44).

Regarding the risk of DM in MS patients, findings across studies are inconsistent. One cohort study found no significant difference in T2DM risk between MS patients and the general population (45), while another large population-based matched cohort study indicated a significantly increased risk of T2DM in MS patients following diagnosis (46). Although our meta-analysis did not find a statistically significant increase in DM risk among MS patients, the underlying mechanisms warrant further investigation. The inflammatory process in MS may induce a systemic inflammatory response, potentially affecting insulin sensitivity and increasing DM risk (47). The relatively high incidence of DM in MS patients might also be attributed to neurological demyelination-related motor dysfunction or side effects from treatments involving adrenocorticotropic hormone and glucocorticoids (48).

This study has several limitations. First, although the total sample size across included studies is large, the number of eligible studies is limited, which may introduce some bias. Second, the literature search was restricted to English-language publications, potentially leading to language bias. Finally, all included studies were retrospective cohort designs, and their findings may be subject to inherent biases associated with this study design.

## Conclusions

In conclusion, current evidence suggests that T2DM is associated with an increased risk of developing MS, while a significant association between MS and an elevated risk of T2DM was not confirmed. Although our meta-analysis indicates an association, this does not establish causality. Clinicians should maintain heightened vigilance for MS when managing patients with T2DM, particularly those presenting with neurological symptoms. Conversely, regular blood glucose monitoring is advisable for MS patients to enable early detection and management of potential DM. Given the limitations of this study, large-scale, multi-center, long-term prospective cohort studies are needed to further clarify the nature of the association between DM and MS.

## Data Availability

The original contributions presented in the study are included in the article/**Supplementary Material**. Further inquiries can be directed to the corresponding author.
