# Supplementary material for: Diabetes mellitus and risk of multiple sclerosis: a systematic review and meta-analysis
Source: Front Endocrinol (Lausanne). 2026 Feb 18;17:1724167. doi: 10.3389/fendo.2026.1724167 (PMC12957073; doi:10.3389/fendo.2026.1724167)
Supplement: Supplementary file 1 [file Table1.docx]

| **Supplementary Material Table 1. Search strategies** | |
| --- | --- |
| Number | Search words |
| #1 | "Multiple Sclerosis"[MeSH] |
| #2 | "multiple sclerosis"[Title/Abstract] |
| #3 | "sclerosis multiple"[Title/Abstract] |
| #4 | "ms multiple sclerosis"[Title/Abstract] |
| #5 | "sclerosis disseminated"[Title/Abstract] |
| #6 | "disseminated sclerosis"[Title/Abstract] |
| #7 | "multiple sclerosis acute fulminating"[Title/Abstract] |
| #8 | OR/1-7 |
| #9 | "Diabetes Mellitus"[MeSH] |
| #10 | "Diabetes Mellitus"[Title/Abstract] |
| #11 | "diabetes mellitus, type 2"[MeSH Terms] |
| #12 | "diabetes mellitus, type 2"[Title/Abstract] |
| #13 | "diabetes mellitus, stable"[Title/Abstract] |
| #14 | "stable diabetes mellitus"[Title/Abstract] |
| #15 | "diabetes mellitus, noninsulin dependent"[Title/Abstract] |
| #16 | "diabetes mellitus, adult onset"[Title/Abstract] |
| #17 | "adult-onset diabetes mellitus"[Title/Abstract] |
| #18 | "diabetes mellitus, adult onset"[Title/Abstract] |
| #19 | "Diabetes Mellitus, Ketosis-Resistant"[Title/Abstract] |
| #20 | "Diabetes Mellitus, Ketosis Resistant"[Title/Abstract] |
| #21 | "Ketosis-Resistant Diabetes Mellitus"[Title/Abstract] |
| #22 | "diabetes mellitus, non insulin dependent"[Title/Abstract] |
| #23 | "diabetes mellitus, non-insulin-dependent"[Title/Abstract] |
| #24 | "non-insulin-dependent diabetes mellitus"[Title/Abstract] |
| #25 | "diabetes mellitus type II"[Title/Abstract] |
| #26 | "NIDDM"[Title/Abstract] |
| #27 | "diabetes mellitus, maturity-onset"[Title/Abstract] |
| #28 | "diabetes mellitus, maturity onset"[Title/Abstract] |
| #29 | "maturity-onset diabetes mellitus"[Title/Abstract] |
| #30 | "maturity onset diabetes mellitus"[Title/Abstract] |
| #31 | "MODY"[Title/Abstract] |
| #32 | "diabetes mellitus, slow-onset"[Title/Abstract] |
| #33 | "diabetes mellitus, slow onset"[Title/Abstract] |
| #34 | "Slow-Onset diabetes mellitus"[Title/Abstract] |
| #35 | "type 2 diabetes mellitus"[Title/Abstract] |
| #36 | "noninsulin-dependent diabetes mellitus"[Title/Abstract] |
| #37 | "noninsulin dependent diabetes mellitus"[Title/Abstract] |
| #38 | "maturity-onset diabetes"[Title/Abstract] |
| #39 | "diabetes, maturity onset"[Title/Abstract] |
| #40 | "maturity onset diabetes"[Title/Abstract] |
| #41 | "type 2 diabetes"[Title/Abstract] |
| #42 | "diabetes, type 2"[Title/Abstract] |
| #43 | "diabetes mellitus, noninsulin-dependent"[Title/Abstract] |
| #44 | "diabetes mellitus, type 1"[MeSH Terms] |
| #45 | "diabetes mellitus, type 1"[Title/Abstract] |
| #46 | "type 1 diabetes"[Title/Abstract] |
| #47 | "diabetes, type 1"[Title/Abstract] |
| #48 | "diabetes mellitus, insulin-dependent"[Title/Abstract] |
| #49 | "diabetes mellitus, insulin dependent"[Title/Abstract] |
| #50 | "insulin-dependent diabetes mellitus"[Title/Abstract] |
| #51 | "diabetes mellitus, juvenile-onset"[Title/Abstract] |
| #52 | "diabetes mellitus, juvenile onset"[Title/Abstract] |
| #53 | "juvenile-onset diabetes mellitus"[Title/Abstract] |
| #54 | "IDDM"[Title/Abstract] |
| #55 | "diabetes mellitus, type i"[Title/Abstract] |
| #56 | "Diabetes Mellitus, Sudden-Onset"[Title/Abstract] |
| #57 | "Diabetes Mellitus, Sudden Onset"[Title/Abstract] |
| #58 | "Sudden-Onset Diabetes Mellitus"[Title/Abstract] |
| #59 | "Type 1 Diabetes Mellitus"[Title/Abstract] |
| #60 | "Diabetes Mellitus, Insulin-Dependent, 1"[Title/Abstract] |
| #61 | "Insulin-Dependent Diabetes Mellitus 1"[Title/Abstract] |
| #62 | "Insulin Dependent Diabetes Mellitus 1"[Title/Abstract] |
| #63 | "Juvenile-Onset Diabetes"[Title/Abstract] |
| #64 | "Diabetes, Juvenile-Onset"[Title/Abstract] |
| #65 | "Juvenile Onset Diabetes"[Title/Abstract] |
| #66 | "Diabetes, Autoimmune"[Title/Abstract] |
| #67 | "Autoimmune Diabetes"[Title/Abstract] |
| #68 | "Diabetes Mellitus, Brittle"[Title/Abstract] |
| #69 | "Brittle Diabetes Mellitus"[Title/Abstract] |
| #70 | "Diabetes Mellitus, Ketosis-Prone"[Title/Abstract] |
| #71 | "Diabetes Mellitus, Ketosis Prone"[Title/Abstract] |
| #72 | "Ketosis-Prone Diabetes Mellitus"[Title/Abstract] |
| #73 | OR/9-72 |
| #74 | "Association"[MeSH] |
| #75 | "Association"[Title/Abstract] |
| #76 | "Associations"[Title/Abstract] |
| #77 | "Risk"[MeSH] |
| #78 | "Risk"[Title/Abstract] |
| #79 | "Risks"[Title/Abstract] |
| #80 | "relative risk"[Title/Abstract] |
| #81 | "relative risks"[Title/Abstract] |
| #82 | "risk relative"[Title/Abstract] |
| #83 | "risks relative"[Title/Abstract] |
| #84 | "Cox"[Title/Abstract] |
| #85 | "Proportional Hazards Models"[Mesh] |
| #86 | "Proportional Hazards Model"[Title/Abstract] |
| #87 | "Hazards Model, Proportional"[Title/Abstract] |
| #88 | "Hazards Models, Proportional"[Title/Abstract] |
| #89 | "Model, Proportional Hazards"[Title/Abstract] |
| #90 | "Proportional Hazard Model"[Title/Abstract] |
| #91 | "Hazard Model, Proportional"[Title/Abstract] |
| #92 | "Hazard Models, Proportional"[Title/Abstract] |
| #93 | "Model, Proportional Hazard"[Title/Abstract] |
| #94 | "Models, Proportional Hazard"[Title/Abstract] |
| #95 | "Proportional Hazard Models"[Title/Abstract] |
| #96 | "Models, Proportional Hazards"[Title/Abstract] |
| #97 | "Cox Models"[Title/Abstract] |
| #98 | "Cox Model"[Title/Abstract] |
| #99 | "Model, Cox"[Title/Abstract] |
| #100 | "Models, Cox"[Title/Abstract] |
| #101 | "Cox Proportional Hazards Models"[Title/Abstract] |
| #102 | "Cox Proportional Hazards Model"[Title/Abstract] |
| #103 | "Hazards Models"[Title/Abstract] |
| #104 | "Hazards Model"[Title/Abstract] |
| #105 | "Model, Hazards"[Title/Abstract] |
| #106 | "Models, Hazards"[Title/Abstract] |
| #107 | "Hazard Model"[Title/Abstract] |
| #108 | "Model, Hazard"[Title/Abstract] |
| #109 | "Models, Hazard"[Title/Abstract] |
| #110 | "Hazard Models"[Title/Abstract] |
| #111 | OR/74-110 |
| #112 | #8 AND #73 AND #111 |

| \| **Supplementary Material Table 2. Newcastle-Ottawa Scale score** \| \| \| \| \| \| \| --- \| --- \| --- \| --- \| --- \| --- \| \| Author (year) \| Selection \| Comparability \| Outcome \| Total score \| Quality \| \| Kang et al 2010 \| ★★★ \| ★ \| ★★ \| 6 \| Moderate \| \| Hou et al 2017 \| ★★★ \| ★★ \| ★★ \| 7 \| High \| \| Jacobsen et al 2024 \| ★★ \| ★★ \| ★★ \| 6 \| Moderate \| \| Palladino et al 2024 \| ★★ \| ★★ \| ★★ \| 6 \| Moderate \| \| Cho et al 2024 \| ★★★★ \| ★★ \| ★★ \| 8 \| High \| \| Nielsen et al 2021 \| ★★★ \| ★★ \| ★★ \| 7 \| High \| \| Brnabic et al 2024 \| ★★ \| ★★ \| ★★ \| 6 \| Moderate \| |
| --- | --- | --- | --- | --- | --- | --- | --- | --- | --- | --- | --- | --- | --- | --- | --- | --- | --- | --- | --- | --- | --- | --- | --- | --- | --- | --- | --- | --- | --- | --- | --- | --- | --- | --- | --- | --- | --- | --- | --- | --- | --- | --- | --- | --- | --- | --- | --- | --- | --- | --- | --- | --- | --- | --- |
|  |
